# Pseudogene accumulation in the evolutionary histories of Salmonella enterica serovars Paratyphi A and Typhi

**DOI:** 10.1186/1471-2164-10-36

**Published:** 2009-01-21

**Authors:** Kathryn E Holt, Nicholas R Thomson, John Wain, Gemma C Langridge, Rumina Hasan, Zulfiqar A Bhutta, Michael A Quail, Halina Norbertczak, Danielle Walker, Mark Simmonds, Brian White, Nathalie Bason, Karen Mungall, Gordon Dougan, Julian Parkhill

**Affiliations:** 1 Wellcome Trust Sanger Institute, Wellcome Trust Genome Campus, Hinxton, Cambridge, CB10 1SA, UK; 2 Laboratory of Gastrointestinal Pathogens, Centre for Infections, Health Protection Agency, 61 Colindale Avenue, London, NW9 5HT, UK; 3 Aga Khan University, Karachi, Pakistan

## Abstract

**Background:**

Of the > 2000 serovars of *Salmonella enterica *subspecies I, most cause self-limiting gastrointestinal disease in a wide range of mammalian hosts. However, *S. enterica *serovars Typhi and Paratyphi A are restricted to the human host and cause the similar systemic diseases typhoid and paratyphoid fever. Genome sequence similarity between Paratyphi A and Typhi has been attributed to convergent evolution via relatively recent recombination of a quarter of their genomes. The accumulation of pseudogenes is a key feature of these and other host-adapted pathogens, and overlapping pseudogene complements are evident in Paratyphi A and Typhi.

**Results:**

We report the 4.5 Mbp genome of a clinical isolate of Paratyphi A, strain AKU_12601, completely sequenced using capillary techniques and subsequently checked using Illumina/Solexa resequencing. Comparison with the published genome of Paratyphi A ATCC9150 revealed the two are collinear and highly similar, with 188 single nucleotide polymorphisms and 39 insertions/deletions. A comparative analysis of pseudogene complements of these and two finished Typhi genomes (CT18, Ty2) identified several pseudogenes that had been overlooked in prior genome annotations of one or both serovars, and identified 66 pseudogenes shared between serovars. By determining whether each shared and serovar-specific pseudogene had been recombined between Paratyphi A and Typhi, we found evidence that most pseudogenes have accumulated after the recombination between serovars. We also divided pseudogenes into relative-time groups: ancestral pseudogenes inherited from a common ancestor, pseudogenes recombined between serovars which likely arose between initial divergence and later recombination, serovar-specific pseudogenes arising after recombination but prior to the last evolutionary bottlenecks in each population, and more recent strain-specific pseudogenes.

**Conclusion:**

Recombination and pseudogene-formation have been important mechanisms of genetic convergence between Paratyphi A and Typhi, with most pseudogenes arising independently after extensive recombination between the serovars. The recombination events, along with divergence of and within each serovar, provide a relative time scale for pseudogene-forming mutations, affording rare insights into the progression of functional gene loss associated with host adaptation in *Salmonella*.

## Background

*Salmonella enterica *serovars Typhi and Paratyphi A (Typhi, Paratyphi A) are human-restricted bacterial pathogens that cause related systemic diseases, known as typhoid, paratyphoid or enteric fever [1]. Together, these pathogens infect more than 25 million people annually worldwide, resulting in > 200,000 deaths [2]. Historically, Paratyphi A was responsible for less than 20% of these infections [2], however Paratyphi A infection rates have been rising, particularly in South East Asia where this serovar is now responsible for 30–50% of enteric fever cases [3-6]. This increase has been associated with rises in antibiotic resistance among paratyphoid infections [3,7,8]. It may also be associated with vaccination against Typhi, which unfortunately provides little cross-protection against Paratyphi A [9,10]. Finished genomic sequence is currently available for two Typhi isolates (recent clinical isolate CT18 and laboratory strain Ty2) and one Paratyphi A isolate (laboratory strain ATCC9150) [11-13].

Typhi and Paratyphi A are unusual among *S. enterica*, as most serovars infect a broad range of host species and cause self-limiting gastroenteritis, while Typhi and Paratyphi A infect only humans and cause systemic disease [14]. The basis for their unusual shared phenotype is unclear. Whole-genome sequence comparisons suggest that the Paratyphi A and Typhi chromosomes are much more closely related at the DNA level than other *S. enterica *serovars. Furthermore the genomes of both organisms harbour a large number of pseudogenes (> 4% of coding sequences in each genome) [11-13] compared to host-generalist relatives such as *S. enterica *serovar Typhimurium (0.9%) or *E. coli *K12 (0.7%).

A recent study showed that the apparent similarity between Paratyphi A and Typhi genome sequences is due to low nucleotide divergence (mean 0.18%) across a quarter of the genome, while the rest of the genome sequences are as divergent as any other pair of *S. enterica *serovars (mean 1.2%) [15]. The study used model-based approaches to demonstrate that this is due to relatively recent convergence via recombination between 23% of the Paratyphi A and Typhi genomes, whose initial divergence occurred around the same time as that of other *S. enterica *serovars. It is possible that this extensive recombination was responsible for the convergence of Paratyphi A and Typhi on a human-restricted lifestyle, however it is also plausible that the serovars followed independent paths to host-restriction and the opportunity for recombination arose after they became isolated together in this shared niche. The direction of recombination cannot be determined, and may have been uni- or bi-directional.

Pseudogenes are coding sequences (CDS) that are putatively inactivated by mutations including nonsense substitutions, frameshifts, or truncation by deletion or rearrangement. Loss of gene function through pseudogene formation and gene deletion appears to be a hallmark of host-restricted pathogenic bacteria compared to their host-generalist relatives [11,13,16-19]. This is likely due to a combination of adaptation (whereby loss of gene function is selected for in the new host) and genetic drift associated with population bottlenecks during or following adaptation to the new niche. It has been reported that Paratyphi A and Typhi share some of their pseudogenes [13], resulting in convergent loss of gene functions which may be associated with adaptation to their shared niche. The genomes of *S. enterica *encode two type III secretion systems (TTSS), which mediate secretion of a range of effector proteins into host cells [20]. Many of these effectors are encoded in *Salmonella *pathogenicity islands 1 and 2 (SPI-1 and SPI-2, reviewed in [20,21]), including several that are pseudogenes in Typhi and/or Paratyphi A. The inactivation of these and other genes involved in interactions between *Salmonella *and host is thought to play a key role in the host adaptation of these serovars [11,13].

Here we report the 4.5 Mbp genome sequence of a recent clinical isolate of Paratyphi A, strain AKU_12601, allowing the first comparative analysis between two Paratyphi A isolates at the whole-genome sequence level. We also present a novel comparative annotation of pseudogenes in all four Paratyphi A and Typhi genomes. This is combined with previously reported divergence data [15] in order to tease apart the roles that recombination and pseudogene formation have played in the genetic and phenotypic convergence of Paratyphi A and Typhi.

## Results and Discussion

### Sequencing the Paratyphi A AKU_12601 genome

The whole genome sequence of Paratyphi A strain AKU_12601 was assembled, finished and annotated as described in the Methods section. The genome consists of a 4,581,797 bp circular chromosome, encoding 4,285 CDS, and a 212,711 bp IncHI1 multidrug resistance plasmid pAKU_1 [EMBL:AM412236] which has been described in detail elsewhere [22]. The AKU_12601 genome was also resequenced using the Illumina Genome Analyzer (Illumina), to a depth of 20-fold coverage. Short reads (35 bp) generated by resequencing were aligned to the finished sequence, which identified five high quality single base discrepancies between the assemblies (see Methods). One was found to be an erroneous base call in the finished sequence following checking of trace files and was corrected prior to EMBL submission. The remaining four bases (6-, 8-, 10-, and 20-fold read depth in Illumina data) may be errors in the Illumina resequencing, or reflect genuine mutations arising during culturing in the laboratory.

#### Data accessions

The finished sequence and annotation of the AKU_12601 genome is available in EMBL under accession FM200053, and the Illumina resequencing data is available under accession ERA000012 .

### Comparison of Paratyphi A strains AKU_12601 and ATCC9150

Comparative analysis revealed the two Paratyphi A genomes to be collinear, with no rearrangements and no acquisitions of phage or other large mobile elements. In contrast, Typhi Ty2 contains an inversion of half the genome between two rRNA operons and large-scale phage variation compared to Typhi CT18 [12]. Several insertion/deletion events and substitutions were identified between the Paratyphi A genomes.

#### Insertions and deletions

A total of 39 insertion/deletion events, including 13 differences in homopolymeric tracts, were identified between AKU_12601 and ATCC9150 (Table 1). Two *IS*10 elements were inserted in AKU_12601, within the *nmpC *gene and a hypothetical pseudogene (SSPA4008a/SPA4318). Six variable number tandem repeats (VNTRs) were identified, including one less tandem copy each of the tRNA-*Gly *and *rrT *RNA genes in AKU_12601.

**Table 1:** Insertion/deletion events between Paratyphi A AKU_12601 and ATCC9150

**Coding effect**	**Gene**	**Mutation**
pseudo-forming	*aidB*	217 bp del
pseudo-forming	*asnB*	1 bp del (homopol)
pseudo-forming	*ccmH*	95 bp del
pseudo-forming	*nmpC*	1338 bp ins (IS10)
pseudo-forming	*pduF*	1 bp del (homopol)
pseudo-forming	*pduG*	171 del
pseudo-forming	*proQ*	7 bp del
pseudo-forming	*rbsC*	1 bp ins (homopol)
pseudo-forming	*rbsR*	1 bp ins (homopol)
pseudo-forming	*rhlB*	2 bp ins
pseudo-forming	SSPA3202	1 bp ins (homopol)
pseudo-forming	*tesB*	352 bp del
pseudo-forming	*wcaA*	1 bp ins (homopol)
pseudo-forming	*yaaJ*	1 bp del (homopol)
pseudo-forming	*yeaG*	1 bp del
pseudo-forming	*yeeO*	1 bp ins (homopol)
already pseudo	SSPA4008a	1338 bp ins (IS10)
coding change	*pduP*	6 bp VNTR
coding change	*rcnA*	12 bp VNTR
coding change	*rmbA*	9 bp del
coding change	SSPA3558a	10 bp del
coding change	*ytfM*	3 bp del
coding change*	SSPA0733,4,5	5770 bp VNTR
intergenic	-	1 bp ins (homopol)
intergenic	-	1 bp ins (homopol)
intergenic	-	3 bp ins (homopol)
intergenic	-	1 bp ins
intergenic	-	1 bp ins
intergenic	-	1 bp ins
intergenic	-	1 bp ins
intergenic	-	1 bp ins
intergenic	-	122 bp VNTR
RNA	*rtT *RNA	175 bp VNTR
rRNA	*rrlC*	1 bp del (homopol)
rRNA	*rrlD*	1 bp ins (homopol)
rRNA	*rrsD*	1 bp ins
sRNA	*csrB*	1 bp ins (homopol)
tRNA	*proL*	7 bp del
tRNA	tRNA-*Gly*	220 bp VNTR

Del – deletion; ins – insertion; homopol – insertion or deletion in homopolymeric sequence; IS10 – *IS*10 transposase insertion; VNTR – variable number tandem repeat. Pseudo-forming – mutation results in formation of a pseudogene in one strain; coding change – mutation results in a change to the translated amino acid sequence but retains the reading frame; * fusion of two genes, retaining the reading frame.

**Figure 1:**
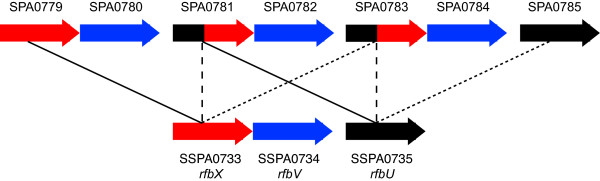
**Tandem repeats in the O-antigen biosynthesis cluster in Paratyphi A ATCC9150**. Bottom row: gene arrangement in Paratyphi A AKU_12601 and Typhi, presumed to be the ancestral form. Top row: gene arrangement in Paratyphi A ATCC9150, apparently resulting from two tandem duplications. Labels give systematic identifiers for the gene sequences in each genome, identical coding sequences are shown in the same colours, identical sequences are joined by lines.

The largest single locus difference between the two genomes occurs within the O-antigen biosynthetic cluster *rfb*, where a 2.7 kb sequence including the 3' end of putative O-antigen transporter *rfbX *(SSPA0733) and two putative glycosyltransferase genes (*rfbV*/SSPA0734 and 5' end of *rfbU*/SSPA0735) is present in three tandem copies in ATCC9150. A single copy of this sequence is present in other *S. enterica *serovars [23], therefore the AKU_12601 sequence is assumed to be the ancestral form. The repeats in ATCC9150 generate two copies of a chimeric coding sequence, combining the 5' end of *rfbU *with the 3' end of *rfbX *(Figure 1). These genes are involved in synthesis and transport of O-antigen [23], but it is unclear whether the increased copy number and chimeric sequences generated by these repeats cause any functional differences in O-antigen expression between ATCC9150 and AKU_12601.

An additional 122 bp sequence was present in AKU_12601 between the *iap *and *ygbF *genes, including two additional copies of a 30 bp repeat sequence present in six copies in ATCC9150. Smaller VNTRs were identified within *pduP *and *rcnA*, resulting in repeats of two and four amino acids respectively in the encoded proteins. VNTRs are useful as genetic markers for typing *Salmonella enterica *serovars, and variability in the *rcnA *VNTR among Paratyphi A isolates has been reported previously [24].

#### Single nucleotide polymorphisms (SNPs)

In addition to insertion/deletion events, 188 SNPs were identified. These include 101 non-synonymous and 51 synonymous SNPs, giving a dN/dS ratio of 0.62, similar to that observed between diverse Typhi strains [25]. While extreme care must be taken in interpreting dN/dS ratios based on the comparison of two closely related genomes [26], this ratio is consistent with some degree of purifying selection in the Paratyphi A population.

#### Differences in pseudogene complements

The Paratyphi A AKU_12601 genome contains 204 pseudogenes, constituting 4.8% of annotated CDSs. Although our comparative analysis revealed very few sequence differences between the two Paratyphi A genomes (188 SNPs, 39 insertion/deletion events), these differences include 22 pseudogene-forming mutations (see Table 2). The mutations include six nonsense SNPs and 16 insertion/deletion events, and were verified by inspecting the capillary sequencing traces and Illumina reads data for Paratyphi A AKU_12601. This suggests that pseudogene-forming mutations are continuing to accumulate in Paratyphi A, as has been observed in Typhi [12,25].

**Table 2:** Inactivating mutations unique to either AKU_12601 or ATCC9150

**Gene**	**Mutation**	**Strain**	**Gene product**
*aidB*	del	AKU_12601	probable acyl Co-A dehydrogenase
*asnB*	1 bp del (homopol)	ATCC9150	asparagine synthetase B
*ccmH*	88 bp del	AKU_12601	cytochrome c-type biogenesis protein H2
*gltJ*	nonsense SNP	AKU_12601	glutamate/aspartate transport system permease
*nmpC*	IS10 ins	AKU_12601	outer membrane porin
*pduF*	1 bp del (homopol)	ATCC9150	propanediol diffusion facilitator
*pduG*	171 bp del	AKU_12601	propanediol dehydratase reactivation protein
*proQ*	7 bp del	ATCC9150	ProP effector
*rbsC*	1 bp ins (homopol)	ATCC9150	high affinity ribose transport protein
*rbsR*	1 bp ins (homopol)	ATCC9150	ribose operon repressor
*rhlB*	2 bp ins	ATCC9150	putative ATP-dependent RNA helicase
SSPA1447	nonsense SNP	AKU_12601	putative oxidoreductase
SSPA3202	1 bp ins (homopol)	AKU_12601	putative lipoprotein
SSPA3581	nonsense SNP	AKU_12601	conserved hypothetical protein
*tesB*	352 bp del	ATCC9150	acyl-CoA thioesterase II
*trpD*	nonsense SNP	AKU_12601	anthranilate synthase component II
*wcaA*	1 bp ins (homopol)	AKU_12601	putative glycosyl transferase
*yaaJ*	1 bp del	ATCC9150	putative amino-acid transport protein
*yeaG*	1 bp del	ATCC9150	conserved hypothetical protein
*yeeO*	1 bp ins (homopol)	ATCC9150	putative inner membrane protein
*yhaO*	nonsense SNP	ATCC9150	putative transport system protein
*yjhW*	nonsense SNP	ATCC9150	putative membrane protein

Insertion, deletion or substitution events identified between Paratyphi A strains AKU_12601 and ATCC9150, causing gene inactivation in one strain. Strain – Paratyphi A strain in which the inactivating mutation occurs; del – deletion; ins – insertion; homopol – variation in homopolymeric sequence.

### Comparison of pseudogenes in Paratyphi A and Typhi genomes

In order to comprehensively investigate the mechanisms of convergent gene loss in Paratyphi A and Typhi, we assembled a comparative table of pseudogenes present in each serovar (Additional file 1). This analysis includes all previously annotated pseudogenes, some additional Typhi pseudogenes suggested previously [13] and some novel pseudogenes identified by manually inspecting Typhi and Paratyphi A sequences for all genes annotated as pseudogenes in any of the AKU_12601, ATCC9150, CT18 or Ty2 genomes (see Methods).

#### Shared pseudogenes

The resulting table includes 66 pseudogenes common to Typhi (strains CT18, Ty2) and Paratyphi A (strains AKU_12601, ATCC9150) (Additional file 1). This is almost double the figure reported previously [13], although many of the additional pseudogenes are remnants of transposase or bacteriophage genes. By aligning the Typhi and Paratyphi A DNA sequences for the shared pseudogenes, we identified shared and independent inactivating mutations (Additional file 1). Contrary to previous reports [13], we found common inactivating mutations in many of the shared pseudogenes.

The functions of most of the shared pseudogenes was discussed by the authors of the ATCC9150 genome study [13] and need not be repeated here. Of particular note, however, 20 of the shared pseudogenes (54% of non-phage/transposase shared pseudogenes) encode secreted or surface-exposed proteins (Table 3), thus are likely to have contributed to convergence upon similar patterns of host interactions. Furthermore, inactivation of different genes in the same pathway will often result in similar loss of function, thus the true contribution of pseudogene formation to phenotypic convergence between Typhi and Paratyphi A is likely underestimated by considering only shared pseudogenes. For example, different members of the *cbi *cluster are inactivated in Typhi and Paratyphi A, which may result in similar inactivation of the cobalamin synthesis pathway [13].

**Table 3:** Pseudogenes shared between Paratyphi A and Typhi

**Class**	**SSPA**	**STY**	**Gene**	**Gene product**	**Div.**
i^	0062a	n/a	-	putative viral protein	-
i^	0255a	n/a	-	putative uncharacterized protein	-
i	1103	1362	-	Pertussis toxin subunit S1 related protein	1.22%
i^	1699a	0971	*sopD2*	*secreted effector protein SopD homolog	1.73%
i^	2014	0610	*silA*	*putative inner membrane proton/cation antiporter	1.08%
i^	2014a	0609a	*cusS*	*putative copper-ion sensor protein	0.18%
i^	3229	4202	-	putative phosphosugar-binding protein	0.14%
i	3640	3800	*cdh*	CDP-diacylglycerol pyrophosphatase	2.32%
i^	3888	4728a	-	putative uncharacterized protein	1.35%
i	*30 transposase/phage genes and gene remnants, details available in Additional file *1				
ii	0097	0113	-	*putative secreted protein	0.25%
ii	0431b	2631	-	putative IS transposase	0.24%
ii	0754a	2275	*sopA*	*secreted effector protein	0.23%
ii	3228	4203	-	putative L-asparaginase	0.14%
ii	3365a	4037	*sugR*	putative uncharacterized protein (SPI-3)	0.14%
iii	0192a	0218	*fhuA*	*ferrichrome-iron receptor precursor	23.95%
iii	0317a	2775	-	putative anaerobic dimethylsulfoxide reductase component	1.79%
iii	0329a	2762	*sivH*	*putative invasin (CS54)	1.17%
iii	0331a	2758	*ratB*	*putative lipoprotein (CS54)	1.67%
iii	0331b	2755	*shdA*	*putative uncharacterized protein (CS54)	2.11%
iii	0621a	2422	*mglA*	*galactoside transport ATP-binding protein	1.09%
iii	0720a	2311	*wcaK*	*putative extracellular polysaccharide biosynthesis protein	1.82%
iii	0756a	2268	*yeeC*	penicillin-binding protein	2.19%
iii	0850a	2166	*fliB*	lysine-N-methylase	3.11%
iii	0943a	1995	-	transposase	4.77%
iii	1014a	1913	*hyaA*	hydrogenase-1 small subunit	0.33%
iii	1220a	1508	-	*putative transport protein	1.31%
iii	1367a	1739	-	putative ribokinase (SPI-2)	1.42%
iii	1531a	1244	*fhuE*	*FhuE receptor precursor	0.96%
iii	1642a	1104	-	*putative secreted protein	1.54%
iii	1820a	0833	*slrP*	*secreted effector protein	1.95%
iii	2045a	0569	*ybbW*	*putative allantoin transporter	1.19%
iii	2301a	0333	*safE*	*probable lipoprotein (SPI-6 fimbrial cluster)	1.52%
iii	3388a	4007	-	putative cytoplasmic protein	1.12%
iii	3636a	3805	-	*putative permease of the Na+:galactoside symporter family	2.42%
iii	3828b	4503	*dmsA*	anaerobic dimethyl sulfoxide reductase chain A	0.22%
iii	3998a	4839	*sefD*	*putative fimbrial protein (SPI-10)	0.18%
iii, iv^1^	1197a	1486	*narW*	respiratory nitrate reductase 2 delta chain	1.74%
iii, iv^2^	2900a	3421	*yhaO*	*putative transport system protein	0.58%
iv^3^	0708	2328	*wcaA*	putative uncharacterized protein	1.35%

(i) Ancestral pseudogenes (shared by virtue of inheritance in inactived gene from a common ancestor); ^ intact in Typhimurium, note that 30 ancestral pseudogenes encoding phage or transposase genes are excluded here but listed in Additional file 1. (ii) Recombined pseudogenes (shared by recombination). (iii) Recent conserved pseudogenes (independent inactivating mutations in each serovar). (iv) Recent strain-specific pseudogenes (pseudogenes in some but not all strains belonging to their respective serovar); ^1^pseudogene in Paratyphi A (both strains) and Typhi Ty2, ^2^pseudogene in Typhi (both strains) and Paratyphi A ATCC9150, ^3^pseudogene in Paratyphi A ATCC9150 and Typhi CT18. SSPA and STY -systematic identifiers in Paratyphi A AKU_12601 and Typhi CT18 respectively; n/a – not annotated. For genes lying in *Salmonella *pathogenicity islands (SPIs) the island is indicated in brackets after the gene product. Div. – nucleotide divergence reported in [15].

### Were pseudogenes shared by recombination?

Recombination has clearly been an important mechanism of convergence between Paratyphi A and Typhi [15]. The accumulation of pseudogenes is a convergent trait evident in these genomes, and shared patterns of pseudogene formation is a likely mechanism for phenotypic convergence. But did recombination contribute to the sharing of pseudogenes?

More than 30% of the pseudogene complements of Typhi and Paratyphi A were shared (Additional file 1), consistent with the possibility that recombination of 23% of the genomes resulted in direct sharing of many of their pseudogenes. We determined whether each pseudogene lay in regions that were predicted to have undergone relatively recent recombination between Paratyphi A and Typhi (sequence divergence < 0.3% between serovars according to [15]) (see Additional file 1). Of all the pseudogenes present in both Paratyphi A AKU_12601 and ATCC9150, 24.3% lie in recently recombined regions; of the pseudogenes present in both Typhi CT18 and Ty2, 25.0% lie in recombined regions. According to [15], 25.6% of genes in CT18 lie in the recently recombined regions.

**Figure 2:**
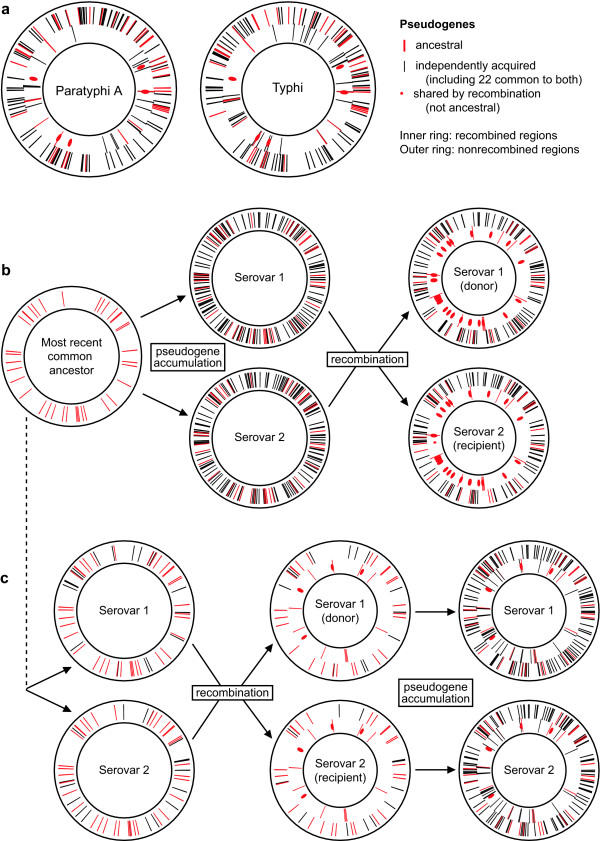
**Scenarios of recombination and pseudogene formation in Paratyphi A and Typhi**. (a) True distribution of pseudogenes in the Paratyphi A AKU_12601 and Typhi CT18 genomes (gene order based on gene co-ordinates in Typhi CT18). (b-c) Distribution of pseudogenes resulting from data simulated under two scenarios, under both of which 40 pseudogenes are inherited from the most recent common ancestor of Paratyphi A and Typhi, and extensive accumulation of pseudogenes occurs before or after recombination of 25% of genes. For ease of simulation, the recombination shown is uni-directional, but bi-directional exchange would result in similar patterns. (b) Scenario 1: 150 additional pseudogenes accumulate in each serovar, followed by recombination. (c) Scenario 2: only 20 additional pseudogenes arise before recombination, after which a further 150 pseudogenes accumulate in each serovar.

These observations are consistent with two scenarios, illustrated in Figure 2: (1) most pseudogenes were inactivated prior to recombination, and recombination was random with respect to the location of pseudogenes (Figure 2b); or (2) most pseudogenes were inactivated after recombination, and these pseudogene-forming mutations were random with respect to recombined regions (Figure 2c). If (1) were true, we would expect that (i) genes that are pseudogenes in one serovar but intact in the other (i.e. serovar-specific pseudogenes) would not lie in recombined regions, and (ii) most pseudogenes in recombined regions would have been shared during recombination, i.e. they would be pseudogenes in both Paratyphi A and Typhi and share common inactivating mutations in both genomes (red circles in Figure 2b). If (2) were true, we would expect that (i) serovar-specific pseudogenes would be distributed randomly with respect to recombined and nonrecombined regions, and (ii) very few pseudogenes would have been shared during recombination, i.e. very few pseudogenes in recombined regions would share inactivating mutations (red circles in Figure 2c).

The distribution of serovar-specific and shared pseudogenes in recombined and nonrecombined regions is shown in Figure 2a and summarised in Table 4. Pearson χ^2 ^tests for each serovar based on this data give non-significant results (*p - value *> 0.2, Table 4), thus there is no evidence of association between shared or serovar-specific pseudogenes and regions of recombination, consistent with scenario (2). More than 20% of serovar-specific pseudogenes lie in recombined regions of each genome (Figure 2a, black lines in inner ring), consistent with scenario (2) whereby serovar-specific pseudogenes are expected to be randomly distributed in the genome of which 23% has been recombined (Figure 2c, black lines in inner ring). These observations are extremely unlikely under scenario (1), which would predict recombination to result in shared but not serovar-specific pseudogenes being present in recombined regions (Figure 2b, inner ring).

**Table 4:** Distribution of serovar-specific and shared pseudogenes in recombined regions

**Distribution**	**Recombined**	**Nonrecombined**	**χ^2 ^test, specific vs. shared**
Typhi-specific	114	39	0.33 (p-value = 0.57)
Paratyphi A-specific	92	24	1.63 (p-value = 0.20)
Shared	46	20	

Pearson χ^2 ^tests were performed separately for each serovar based on the two-way contingency table obtained from the respective serovar-specific row and shared row.

We found only 18 pseudogenes in recombined regions harboured the same inactivating mutations (red lines and circles in inner rings, Figure 2a), less than 20% of pseudogenes in the recombined regions of each genome (Additional file 1). As illustrated in Figure 2, this is consistent with scenario (2) but not scenario (1), which would predict that most pseudogenes lying in recombined regions would be shared by virtue of recombination and therefore carry the same inactivating mutations (red circles in Figure 2).

The patterns of pseudogene distribution we observe therefore suggest that the majority of pseudogenes present in the extant genomes of Paratyphi A and Typhi accumulated after the recombination of 23% of their genomes. Whether this relationship is causal though, remains to be proven. The acceleration of pseudogene formation is most likely due to a combination of host-adaptation and genetic drift associated with a population bottleneck in the new human-restricted niche. However whether the extensive recombination between Typhi and Paratyphi A resulted in, or resulted from, human-restriction of the two organisms, is unknown. It is plausible that host-restriction occurred independently in Typhi and Paratyphi A, providing both (a) an opportunity for recombination soon after they became isolated together in this shared niche, and (b) a trigger for accelerated pseudogene formation. Alternatively, a chance recombination event may have led to host-restriction of both organisms. It has been noted that recombination between Paratyphi A and Typhi involved sharing of intact serovar-specific or rare genes, resulting in many more shared rare genes than would be expected otherwise [15] and presumably promoting the sharing of novel functions. It is plausible therefore that recombination between Paratyphi A and Typhi led to a combination of gene acquisition and loss-of-function resulting in restriction to the human host, bestowing upon these serovars a unique and novel genetic profile that contributed to host restriction and the ability to cause systemic infection. Such an event would likely set Paratyphi A and Typhi on a similar trajectory of host adaptation and associated population bottlenecks, which might account for their similar profiles of rapid accumulation of pseudogenes through adaptive selection and genetic drift.

### Tracing pseudogene formation in the evolutionary histories of Paratyphi A and Typhi

**Figure 3:**
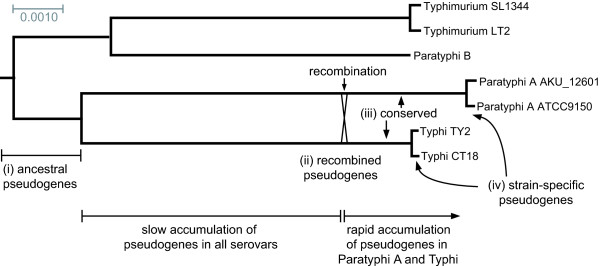
**Pseudogene formation in the evolutionary histories of Paratyphi A and Typhi**. Phylogenetic tree based on multiple alignments of all nonrecombined genes as defined in [15], rooted using *S. bongori *and *E. coli *as outgroups. Scale bar is nucleotide divergence. The timing of the recombination between Paratyphi A and Typhi is an approximation inferred from published divergence data [15]. Group (i) pseudogenes were inactivated prior to the divergence of Paratyphi A and Typhi, some are also inactivated in Typhimurium and Paratyphi B; following their divergence Paratyphi A and Typhi likely accumulated few additional pseudogenes; during the recombination of 23% of their genomes (direction of transfer unknown) 18 pseudogene sequences were shared between Paratyphi A and Typhi, including five non-ancestral pseudogenes (group ii); many pseudogenes were formed during a period of accelerated pseudogene accumulation in both serovars, including most group (iii) pseudogenes; pseudogenes continue to accumulate in individual sub-lineages after the most recent common ancestor of each serovar (group iv).

The recombination described between Paratyphi A and Typhi provides a rare marker of relative time in the evolutionary histories of these organisms. The recombination was discovered by analysing the distribution of nucleotide divergence levels between different regions of the two genomes, which clearly identified a distinct sub-population of low divergence corresponding to the recombined regions (mean 0.18% compared to genome average of 1.2%) [15]. Although not providing a precise measure of age, this suggests that the recombination event happened approximately 15% (0.18/1.2 = 0.15) as long ago as the initial divergence of Paratyphi A, Typhi and other *S. enterica *serovars. This implies that recombination occurred well before the most recent common ancestors of each serovar (see Figure 3), and thus prior to the last population bottlenecks in the Paratyphi A and Typhi populations.

We divided the pseudogenes into distinct categories with different relative ages (Additional file 1): (i) ancestral pseudogenes (shared pseudogenes inactivated prior to the divergence of Paratyphi A and Typhi), (ii) recombined pseudogenes (shared pseudogenes in recombined regions, with shared inactivating mutations assumed to have arisen after initial divergence), (iii) recent conserved pseudogenes (including serovar-specific pseudogenes, and shared pseudogenes containing different inactivating mutations in Paratyphi A and Typhi; the majority of these are expected to have become pseudogenes after recombination) and (iv) recent strain-specific pseudogenes (pseudogenes in some but not all strains belonging to their respective serovar). Table 3 summarises the shared pseudogenes in each category (excluding ancestral transposase/phage gene remants) and Figure 3 shows their approximate timing overlaid on a phylogenetic tree of *S. enterica *serovars. Note that some serovar-specific pseudogenes (group iii) will likely be shown to be strain-specific (group iv) as more strains are sequenced (see below).

#### Ancestral pseudogenes

The inactivating mutations in group (i) pseudogenes are assumed to have been inherited by Paratyphi A and Typhi from a common ancestor (Figure 3). Alternatively some may have been exchanged between Paratyphi A and Typhi soon after their divergence from other *S. enterica*. Either way, these pseudogenes were among the earliest to arise in the evolutionary history of Paratyphi A and Typhi, thus their inactivation has been well tolerated in these serovars (most have also accumulated secondary mutations). This is unsurprising for the majority of ancestral pseudogenes which are insertion sequence (*IS*) transposase and phage genes/fragments. However the inactivation of seven genes known to be functional in Typhimurium and other *Salmonella*, in particular those that are secreted or surface exposed (Table 3), is likely to have had significant functional impact including potential modulations of host interactions. It is also possible that the loss of these genes had little effect on the pathogenic potential of Paratyphi A and Typhi and that they had classic *S. enterica *host-generalist lifestyles until much later on. However the best described of these seven co-inherited pseudogenes is the secreted effector protein *sopD2*, which in Typhimurium is involved in host interactions and virulence [27] and therefore constitutes a plausible candidate for an early modulator of host interactions in Paratyphi A and Typhi.

#### Pseudogenes shared by recombination

Group (ii) contains five recombined pseudogenes (Table 3), which display 0.14–0.25% nucleotide divergence between the two serovars compared to a genome average of 1.2% and thus were likely exchanged long after the initial divergence of Paratyphi A and Typhi (Figure 3). One of these encodes an *IS *transposase, leaving four candidates for convergence via shared gene inactivation directly attributable to recombination. These include the secreted effector protein *sopA*, which mimics mammalian ubiquitin ligase and is recognized and degraded by the human ubiquitination pathway [28]. It is necessary for virulence in both murine systemic infections and bovine gastrointestinal infections by Typhimurium [29,30], thus is clearly important for interactions between *Salmonella *and mammalian hosts. The loss of this gene in Paratyphi A and Typhi may therefore have been an important factor in the restriction or adaptation of these serovars to the human systemic niche. *SopA *is also a pseudogene in the sequenced Paratyphi B strain SPB7 [EMBL:CP000886], although this is difficult to interpret as it is unclear whether this strain is of the systemic or enteric pathotype (negative for tartrate fermentation, but also *sopE*-negative using PCR described in [31]). The other genes are putative uncharacterised SPI-3 protein *sugR*, and two genes not annotated previously in the ATCC9150 genome – putative secreted protein SSPA0097 (interrupted by *IS*200 insertion) and putative L-asparaginase protein SSPA3228 (truncated at both ends by deletions).

#### Recent pseudogenes: convergence after recombination

In addition to > 100 pseudogenes specific to each serovar, group (iii) includes 22 shared pseudogenes containing different inactivating mutations in Paratyphi A and Typhi (Table 3). While it is possible that some of those lying outside recombined regions may have been present prior to recombination, we propose that most of these mutations arose in the period of rapid pseudogene accumulation after recombination. These pseudogenes are examples of convergent gene loss through independent mutation, and are therefore good candidates for involvement in adaptation to the human host. They include only one transposase gene, the remainder being genes of known or putative function, many of which have been implicated in host interactions in serovar Typhimurium (e.g. *fhuA, fhuE, shdA, ratB, sivH*) [13,32]. Two of the independently acquired pseudogenes, both members of fimbrial clusters lying in *Salmonella *pathogenicity islands (*safE *in SPI-6, *sefD *in SPI-10), were not identified in previous pseudogene comparisons [13].

It is not possible to distinguish whether there has been adaptive selection against the activity of these genes in Paratyphi A and Typhi, or simply shared tolerance for their inactivation. For example, it has been noted [13] that three of these genes (*shdA, ratB *and *sivH*, part of the 25 kbp pathogenicity island CS54 [32]) are involved in intestinal colonization and persistence, which does not occur in typhoid or paratyphoid infection. However we cannot distinguish whether the independent inactivation of these genes in each serovar is due to selection against colonization of the intestine (which may stimulate host immune responses), or genetic drift since intestinal colonization is not required to sustain a systemic infection.

#### Ongoing accumulation of strain-specific pseudogenes

A recent comparative analysis of whole-genome variation in 19 Typhi strains inferred that their last common ancestor harboured only 180 pseudogenes, while individual isolates had each accumulated at least 10–28 additional pseudogenes since their divergence from that ancestor [25]. The number was predicted to be an underestimate, as it did not take into account pseudogene formation via insertion/deletion of one or two nucleotides which would introduce frameshifts. In our comparison of the AKU_12601 and ATCC9150 genomes we found 22 mutations resulting in strain-specific pseudogene formation (10–12 per strain, Table 2), and we predict that future comparative analyses of additional strains will uncover further examples of recently acquired strain-specific pseudogenes. These strain-specific pseudogenes must have arisen since the most recent common ancestors of the respective Paratyphi A and Typhi populations and are therefore more recent than those that are conserved within the serovars (see Figure 3). It is interesting to note that three genes were identified with strain-specific mutations in one serovar and independent mutations in the other serovar (see Additional file 1). This may provide the opportunity for ongoing convergence between sub-lineages of the Typhi and Paratyphi A populations as each serovar continues to evolve and adapt.

## Conclusion

The Paratyphi A AKU_12601 genome sequence presented here allowed the first whole-genome comparison between Paratyphi A strains. By comparing the annotation of pseudogenes in these Paratyphi A genomes and the two finished Typhi genomes CT18 and Ty2, we were able to identify novel examples of pseudogenes that are shared between these human-adapted serovars. Paratyphi A and Typhi have each undergone a parallel, rapid accumulation of pseudogenes after extensive recombination of their genomes.

Although Paratyphi A and Typhi share 27 pseudogenes over and above those inherited in inactive form from a common ancestor, only five were shared via recombination while 22 are the result of more recent convergence through independent adaptive mutation. Therefore recombination and pseudogene formation have played largely independent roles in the genetic convergence of Paratyphi A and Typhi.

The recombination between Paratyphi A and Typhi enabled us to identify different groups of pseudogenes that have arisen in these genomes at different points in their evolutionary histories. This implicates loss-of-function of a few genes in early restriction to the human host (ancestral pseudogenes including *sopD2*) and some in subsequent convergent adaptation to the new niche (conserved and in particular shared conserved pseudogenes including *shdA, ratB, sivH*). Pseudogenes shared by recombination (e.g. *sopA*) may have contributed to host-restriction or host-adaptation.

While the analysis presented here considers only Paratyphi A and Typhi, there are other examples of human-adapted *S. enterica *serovars, including Sendai, Paratyphi C and the systemic pathovar of Paratyphi B. It can be expected that as genome sequences for these become available, comparative analysis may yield further insights into their mechanisms of host adaptation. However the occurrence of relatively recent recombination between Paratyphi A and Typhi has afforded a unique insight into the order of events and mechanisms involved in their convergent evolution, a scenario which has likely been played out in many other host-adapted bacteria.

## Methods

### Sequencing of AKU_12601

Paratyphi A strain AKU_12601 was isolated from a Pakistani paratyphoid patient in Karachi, Pakistan in 2002. The whole-genome shotgun consisted of 83,857 paired-end reads from libraries of 2 to 2.8 kb in pUC19, 5 to 6 kb in pMAQ1, and 6 to 9 kb in pMAQ1, giving 9.8-fold coverage. A scaffold was produced using 1,180 paired-end reads from a 20- to 30-kb library in pBACe3.6. The whole genome sequence was finished to standard criteria [33], using 9,879 directed sequencing reads. The sequence was annotated, and the annotation was manually curated using Artemis software [34] as previously described [33]. The sequence includes both the chromosome, presented here, and the 212,711 bp IncHI1 multidrug resistance plasmid pAKU_1 which has been described in detail elsewhere [22]. AKU_12601 was also resequenced using the Illumina Genome Analyzer (Illumina), with 3,191,127 single-end 35 bp reads providing 21.9-fold coverage of the chromosome.

### Sequence comparisons

Maq [35] was used to map Illumina/Solexa 35 bp reads to the finished AKU_12601 sequence and identify potential errors (reported as SNPs by Maq using default parameters). Capillary traces were manually inspected for the five loci at which SNPs were reported by Maq with consensus base quality > 20 and read depth > 5.

Pairwise whole-genome sequence comparisons were generated with blastn and visualized using ACT [36]. Insertions, deletions and nucleotide substitutions between the collinear Paratyphi A AKU_12601 and ATCC9150 genomes were identified using diffseq (EMBOSS [37]).

### Comparison and annotation of pseudogenes

In order to compare annotated genomes of Paratyphi A AKU_12601 [EMBL:FM200053] and ATCC9150 [EMBL:CP000026], Typhi CT18 [EMBL:AL513382] and Ty2 [EMBL:AE014613] with Typhimurium LT2 [EMBL:AE006468], pairwise whole-genome sequence comparisons were generated with blastn and visualized using ACT [36]. Every gene annotated as a pseudogene in any Typhi or Paratyphi A genome was manually inspected in all five genomes, and its pseudogene status in each genome reassessed. All pseudogenes identified in this way are present in the AKU_12601 genome annotation, although many such genes are not annotated in all of ATCC9150, CT18 and Ty2. For coding sequences found to be a pseudogene in more than one serovar, multiple alignments were used to determine whether the same or independent inactivating mutation(s) were present in the different serovars.

### Data simulation

An initial set of 40 genes were selected at random to represent ancestral pseudogenes. Additional sets of 20 and 150 genes were selected at random for each of two serovars, to represent pseudogenes that accumulated after initial divergence of the serovars (sampling with replacement). The same random sets of pseudogenes were used to simulate both scenarios, with only the timing varying (set of 150 pseudogenes arising before or after recombination). To simulate uni-directional recombination events depicted in Figure 2, serovar 2 pseudogenes lying in recombined regions were replaced with serovar 1 pseudogenes lying in recombined regions. All genes were selected at random from 4600 annotated in Typhi CT18, and their status as recombined or nonrecombined was taken directly from the table of Typhi genes provided in [15].

### Phylogenetic analysis

Nucleotide sequences for genes that have not undergone recent recombination between Typhi and Paratyphi A (according to the table provided in [15]) were extracted from the CT18 genome sequence using Artemis. Homologous sequences in other genomes were identified using blastn, top scoring gapped sequence alignments for each genome were assembled into a single multiple alignment for each gene using Mview [38], which were then concatenated. The analysis included Typhimurium (strains LT2, SL1344) and *S. enterica *serovar Paratyphi B SPB7 [EMBL:CP000886], *S. bongori *and *E. coli *K12 [EMBL:U00096] were included as outgroups to root the tree. The *S. bongori *and Typhimurium SL1344 sequences are available from the Wellcome Trust Sanger Insitute [39]. MrBayes [40] was used to fit a phylogenetic model to the concatenated multiple alignment of all (nonrecombined) genes (GRT+Γ model, 200,000 iterations), Figure 3 shows the consensus tree.

## Authors' contributions

KH performed comparative annotation, sequence analysis and phylogenetic analysis of the genomes and drafted the manuscript. NRT participated in annotation and GCL participated in comparative annotation and analysis. RH and ZB isolated AKU_12601 and provided DNA for sequencing. MQ, NB and HN participated in sequencing, while DW, MS, BW and KM participated in finishing the AKU_12601 chromosome sequence. JP, GD and JW conceived of the study, participated in its design and coordination and helped to draft the manuscript. All authors read and approved the final manuscript.

## Supplementary Material

Additional file 1 **Pseudogenes present in Paratyphi A AKU_12601, ATCC9150 or Typhi CT18, Ty2.** Details of all pseudogenes present in finished genomes of Paratyphi A or Typhi, including gene identifiers in all four genomes, nucleotide divergence between Paratyphi A and Typhi, and classification into different classes: pseudogenes in both Paratyphi A and Typhi (ancestral, shared by recombination, independent mutations), pseudogenes in either Paratyphi A or Typhi, and genome-specific pseudogenes. Click here for file
